# Carotid Artery Stenosis: A Look Into the Diagnostic and Management Strategies, and Related Complications

**DOI:** 10.7759/cureus.38794

**Published:** 2023-05-09

**Authors:** Aqsa Ismail, Shivani Ravipati, Diana Gonzalez-Hernandez, Hashim Mahmood, Alizay Imran, Eduardo J Munoz, Saad Naeem, Zain U Abdin, Humza F Siddiqui

**Affiliations:** 1 Department of Medicine, United Medical and Dental College, Karachi, PAK; 2 Department of Medicine, Dr. Pinnamaneni Siddhartha Institute of Medical Sciences and Research Foundation, Vijayawada, IND; 3 Department of Medicine, Universidad Nacional Autónoma de México (UNAM), Mexico City, MEX; 4 Department of Medicine, University College of Medicine and Dentistry, University of Lahore, Lahore, PAK; 5 Department of Surgery, Windsor University School of Medicine, Chicago, USA; 6 Department of General Medicine, Montemorelos University, Montemorelos, MEX; 7 Department of Internal Medicine, Faisalabad Medical University, Faisalabad, PAK; 8 Department of Internal Medicine, Punjab Social Security Hospital, Faisalabad, PAK; 9 Department of Medicine, District Head Quarters Hospital, Faisalabad, PAK; 10 Department of Medicine, Jinnah Sindh Medical University, Karachi, PAK

**Keywords:** duplex ultrasound (dus), asymptomatic carotid stenosis, carotid stenosis, carotid artery stenting (cas), carotid endarterectomy (cea)

## Abstract

Carotid stenosis (CS) is a buildup of atherosclerotic plaque within the artery leading to a wide range of symptoms, from mild symptoms, including blurred vision and confusion, to much more life-threatening presentations, including paralysis due to stroke. The presentation is insidious, with symptoms exhibiting predominantly at severe stenosis; hence the emphasis is placed on the importance of early diagnosis, treatment, and lifestyle modifications. CS is seen undergoing almost the same pathogenesis of any atherosclerotic plaque formation, from endothelial damage of the artery lumen to the formation of a fibrous cap with a foam cell, lipid-filled core. The findings of our review article were consistent with the recent literature, depicting that comorbid hypertension, diabetes, and chronic kidney disease (CKD), and lifestyle aspects, including smoking and diet, played the most salient role in plaque development. Among several imaging modalities, duplex ultrasound (DUS) imaging is the widely preferred method in clinical practice. Carotid endarterectomy (CEA) and carotid stenting are the primarily advocated procedures for symptomatic severe stenosis, with similar long-term outcomes. Although, earlier clinical trials showed promising results in mitigating the risk of stroke among asymptomatic severe CS with surgical intervention. However, recent advancements have shifted the focus to medical management alone due to comparable results among the asymptomatic population. Both surgical and medical regimens are beneficial in treating patients, but it is still an ongoing debate as to which is predominantly superior. The currently advancing trials and research will help elucidate definitive guidelines. However, the massive impact of lifestyle modifications advocates some degree of individualized multidisciplinary management strategies.

## Introduction and background

**Figure 1:**
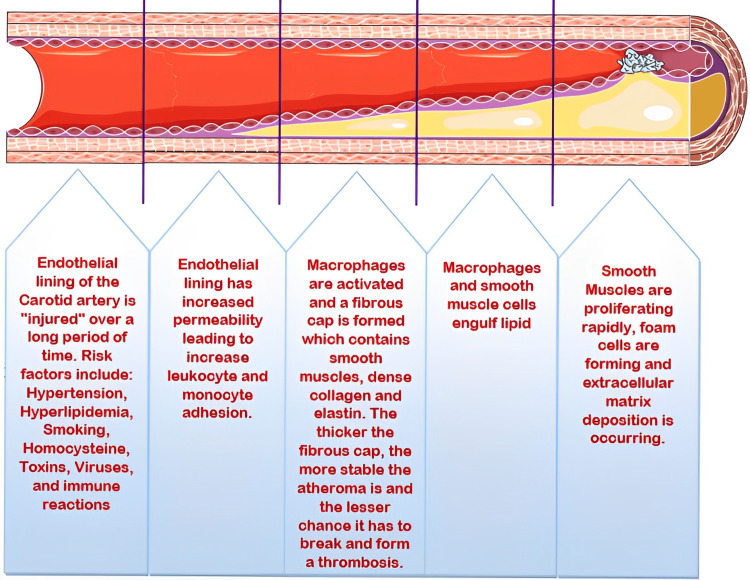
Pathogenesis of carotid artery stenosis. Source: Parts of the figure were drawn using pictures from Servier Medical Art. Servier Medical Art by Servier is licensed under a Creative Commons Attribution 3.0 Unported License (https://creativecommons.org/licenses/by/3.0/).

Carotid stenosis (CS) is defined as the narrowing or blockage of the carotid arteries caused by plaque buildup, which may lead to an increased risk of cerebrovascular events. To understand how CS causes its effect, it is imperative to understand the carotid arteries’ anatomical significance [1, 2]. The common carotid artery exists in pairs, with the right branch originating from the brachiocephalic trunk (which itself branches from the arch of the aorta giving off the right subclavian artery and the right common carotid) and the left common carotid artery originating from the aortic arch directly. The carotids run parallel to each other, placed laterally to the trachea and esophagus inside the deep cervical fascia. The two carotid arteries, covered by the carotid sheath, run medially to the internal jugular vein and anterior to the vagus nerve. Within the lower neck, the carotid artery is present alongside a few clinically important structures, including the larynx, pharynx, and a more superiorly located thyroid gland. At the level of the omohyoid muscle, the artery is covered by the superficial and deep cervical fascia, along with the sternohyoid, sternocleidomastoid, and sternothyroid muscles, with a superficial cover by the platysma muscle. Slightly superior to the omohyoid is a very important clinical correlation where the sternocleidomastoid branch of the superior thyroid artery crosses the carotid with a superficially placed nerve loop called the ansa cervicalis. The common carotid further branches off into the external and internal carotid arteries. The external carotid artery branch arises at the intervertebral disc level of C3-C4, being placed anteromedial to the internal carotid artery. The external carotid artery branch will give off eight main branches supplying to various head and neck regions. The internal carotid artery arises at the common carotid artery bifurcation, the superior border of the laryngeal cartilage, forming the anterior and middle cerebral arteries, hence vital in forming the circle of Willis present at the base of the cerebral hemispheres. The ophthalmic artery goes on to supply the inner layers of the retina and parts of the orbit, meninges, upper nose, and face [3-6]. The base of the internal carotid artery is dilated. This specific dilation is known as the carotid sinus, which has vital baroreceptors critical in relaying information about the arterial blood to the hypothalamus via the glossopharyngeal nerve. Also vital in relaying chemical information about the arterial blood to the respiratory centers is the carotid body, which is placed posterior to the carotid bifurcation, also innervated by the glossopharyngeal nerve [7-9]. A closer look at the exact pathogenesis of carotid plaque formation reveals how carotid artery obstruction may lead to severe CS symptoms (Figure 1). The occlusion of the internal carotid artery mainly results from a thrombotic event, with embolization and other hemodynamic-related issues, mainly atherosclerosis and hypertension playing a role in its occlusion. A condition known as carotid territory stroke may also occur due to an acute occlusion, causing cerebral ischemia leading to neuronal death, which may prove fatal if not treated early [2, 10, 11, 12]. Hemodynamic insufficiencies may also occur as a result of cerebral perfusion interference, including conditions such as hypotension, volume loss, and heart failure [13, 14]. 

Symptomatic CS is rapidly becoming a focal point in current cardiology practices worldwide. With worsening diets and sedentary lifestyles, the risk of plaque buildup in the carotid artery is at an all-time high [15]. Patients with advanced CS may present with symptoms of stroke, including drooping of one side of the face, slurred speech, and loss of motor or sensory function. According to a 2019 study investigating the relationship between the presence of CS and stroke prevalence, around half (50%) of the 2707 patients in the study experienced some sort of an ischemic event. These patients were at a much higher risk of either developing or already presenting with hypertension, hypercholesterolemia, diabetes, and ischemic heart disease. Of the 2707 patients examined, 99 (7.9% [6.3-9.5]) developed symptomatic carotid stenosis [16]. CS is classified as mild, moderate, or severe, with the percentage of blockage directly correlating to the classification scale. The latest research shows that although CS may vastly increase the chances of stroke, its prevalence is not as common in the general population. The data on the prevalence of CS has divided the presentation of the condition within the general population into men and women, with the former showing a prevalence of 4.2% and the latter showing a mere 3.4% (p < 0.001) [17]. Although CS is rare in adults under the age of 50 and low in prevalence in patients over 50, the disease is closely tied to higher mortality rates [17]. The estimated prevalence of severe CS (≥70%) is 0.1 to 3.1%, which is directly proportional to age, with an attributable stroke risk of 0.7%. CS is attributable to 90% of carotid revascularizations performed in the United States [18]. A recent study has shown that patients presenting with 50-99% internal CS showed a mortality rate of 19% (213 of the 1121 patients) within a 6-96 month follow-up range [19]. In order to diagnose a patient for CS, the doctor will perform both a physical exam and order investigations to make a definitive diagnosis. On a thorough physical examination, a swooshing sound, also known as a carotid bruit, will be evident with the help of a stethoscope over the carotid region. Further tests may be done to help rule out stroke symptoms, such as memory, strength, and speech tests [19]. In order to diagnose CS, the current gold standard investigation is a noninvasive duplex ultrasound (DUS), complemented by both computed tomography angiography and magnetic resonance angiography to assess the severity of plaque buildup within the artery. Research is ongoing to develop specific biomarkers to help detect attributes associated with CS, such as plaque inflammation, plaque tissue stiffness, or neovascularization of plaque [20]. Treatment modalities differ according to the type of CS, the level of luminal blockage, and if the patient has already suffered from a stroke. Symptomatic CS patients are extremely medically unstable and require immediate intervention, with invasive treatment being the gold standard, including either carotid endarterectomy (CEA) or carotid angioplasty and stenting [21, 22]. Treatment modalities for asymptomatic carotid stenosis (ACS) patients include medications such as anti-hypertensive, anti-hyperlipidemias (mainly statins), and aspirin. Dietary and lifestyle modifications are also essential in the treatment of both symptomatic and ACS [22, 23]. This review aims to present research and guidelines from major journals presenting information about CS. Literature does exist summarizing the diagnostic and treatment modalities of CS; however, this review aims to encompass the entire condition, orating everything there is about this disease and allowing medical professionals to take a three-dimensional approach toward both patient care and outcome. Not only will this help accomplish the best possible result in achieving the proper diagnosis, but it will also help to work towards much better patient outcomes and mitigate the global disease burden. 

## Review

Risk factors

CS can be a serious health risk and is more common in older people. Age can cause changes in the structure and function, such as decreased stiffness and elasticity. A meta-analysis performed utilizing 59 studies conducted over 21 countries showed a drastic increase of 85% in carotid artery thickening and plaque formation among people aged 50-59 years from the years 2000 to 2020 [24]. Risk factors such as male sex, older age, hypertension, smoking, and alcohol use were found to be strongly linked to asymptomatic carotid stenosis (ACS) in the analysis of variables performed in Korea. The total number of participants in this study was 3330, with a combined median age of 70.0 years (range: 50-100 years). A total of 1.1% of people overall were diagnosed with ACS. Males and females had an ACS prevalence of 1.9% and 0.5%, respectively. Age increased the frequency of CS in both sexes; however, males experienced ACS four times more frequently than females (p ≤ 0.001). The multivariate analysis identified age, hypertension, and smoking (whether a current or former smoker) as independent risk factors for the development of ACS [25]. The development of atherosclerosis can start in childhood. Risk factors such as smoking, hypertension, dyslipidemia, obesity, and the presence of certain disorders like diabetes mellitus and Kawasaki disease (KD) have an impact on the early stages and advancement of atherosclerosis in young people [26]. Race has a substantial impact on the occurrence of clinically severe CS. The highest rates of CS are found in Native Americans and Caucasians, while Asian women and African American men appear to have the lowest rates [17]. A study of 669 ischemic stroke patients used a validated meal frequency questionnaire to evaluate the glycemic index (GI) and glycemic load (GL) in patients. Carotid atherosclerotic stenosis was assessed. Traditional risk variables were assessed, including neutrophil to lymphocyte ratio (NLR), fasting plasma glucose, hemoglobin A1C, total cholesterol, triglycerides, low-density lipoprotein cholesterol (LDL-C), high-density lipoprotein cholesterol (HDL-C), C-reactive protein, and homocysteine. Researchers investigated the relationship between GI/GL and the risk variables for cardiovascular disease (CVD) and CS. In these Chinese cerebral infarction patients, higher GI, GL, and HbA1c were positively correlated with a higher degree of CS, particularly in younger patients and women. The risk of CVD is higher in people with chronic kidney disease (CKD) [27]. Atherosclerosis and carotid arteriosclerosis are more prevalent in CKD. Even in CKD patients with a relatively slight decline in glomerular filtration rate (GFR), the arterial stiffening is prominent, but arterial thickness is only noticeable in more advanced stages [28]. In another study, it was shown that a family history of stroke and coronary heart disease (CHD) were significantly associated with the presence of CS, independent of conventional risk factors. Sibling history of stroke or CHD was a stronger risk factor than parental history, and the association was stronger in people with more affected relatives, regardless of the family size. These findings imply that common genetic and environmental factors influence the risk of CAS and that carotid atherosclerosis and CHD share several genetic variations [29]. Three independent risk variables for restenosis were male sex, smoking, and hyperlipidemia. Thus, it is vital to monitor hyperlipidemia, and regular ultra-sonographic follow-up is advised, particularly for male patients who smoke now or in the past [30]. Stroke is a growing public health burden since it can lead to long-term disability. As a result, emphasizing prevention is crucial. Treatment of modifiable risk factors, such as arterial hypertension, the main modifiable contributor to stroke, is the main objective to accomplish [31]. A higher level of high-sensitivity C-reactive protein (hsCRP) marker for systemic inflammation has been associated with a greater risk of CS. High levels of hsCRP may also indicate chronic inflammation, which contributes to atherosclerosis and arterial wall plaque formation [32]. In addition, high fibrinogen levels are linked to increased carotid stasis risk. Fibrinogen is an essential component in atherosclerotic plaque formation and maintenance. It can also lead to stroke-causing thrombosis. These high fibrinogen levels are also linked to hypertension and smoking [33]. Sleep apnea and snoring have also been identified as risk factors for CS [34, 35].

Signs and symptoms

It is becoming increasingly apparent that mobility and balance issues are linked to ACS. According to the 2021 study's findings, advantages to balance and mobility problems may start to wane six weeks after carotid artery surgery. This may suggest that ACS is a risk factor for aging-related mobility impairment that can be modified [36]. Plaque rupture and thromboembolism, rather than hemodynamic failure, are the most frequent causes of stroke caused by CS. Clinically speaking, this manifests as a stroke, transient ischemic attack (TIA), or amaurosis fugax. The term "amaurosis fugax" is used when the symptoms are transient. Due to the strong correlation between carotid plaque rupture and thromboembolism as the mechanism, this distinctive stroke pattern is also known as the "carotid signature" or "carotid embolic pattern." When the pattern is present, but the typical cervical CS is not identified, an atherosclerotic lesion more proximally or distally on the ipsilateral side is almost certainly to blame. The most typical symptoms include dysarthria, contralateral numbness, and weakness of the hand, arm, face, or leg on the opposite side of the body [37]. A thromboemboli lodged in a significant cerebral vessel causes abrupt large vascular occlusion (LVO), the most severe manifestation of carotid artery disease. LVO mainly presents as sudden aphasia, left hemiparesis, right gaze deviation, right middle cerebral artery syndrome (right MCA syndrome) or left MCA syndrome, right hemiparesis, left gaze deviation, and aphasia. A third typical manifestation of CS is monocular vision loss brought on by retinal embolism, which can be temporary or permanent. The CS patient has multiple self-resolving episodes of monocular vision loss addressed as amaurosis fugax. TIA with hemodynamic element presents as hemodynamic stress when the patient stands from a seated position. The patient's contralateral arm and leg will typically shake on standing. Contrarily, orthostatic lightheadedness or dizziness is more frequently caused by other conditions and is not a distinctive sign of carotid artery disease [37]. For identifying clinically significant CS, the carotid bruit has moderate significance. Although the sensitivity is low, the specificity is excellent [38]. Independent of recognized vascular risk factors for vascular cognitive impairment, ACS is linked to cognitive impairment and presents as mild-to-moderate impairment in learning and memory, motor coordination, and processing speed [39].

Diagnostic modalities

Efficient diagnosis and classification of CS into first-degree CS <50% stenosed artery, second-degree CS 51-69% stenosed artery, or third-degree CS 70-99% stenosed artery is one of the crucial steps for proper management [40]. Scientific research and technological advancements have allowed a significant improvement in diagnostic methods. However, the diagnostic process may differ amongst symptomatic and asymptomatic patients [41]. Digital subtraction angiography (DSA) has been the gold standard for verification of the severity of stenosis. A high-contrast illustration of the vessel is achieved by subtracting two X-ray images, one prior to administering contrast and one after that. DSA has also been shown to be convenient in prognosing the cerebral hyperperfusion phenomenon (HPP) with up to 75% sensitivity and 100% specificity [20]. MRI is commonly utilized to obtain high-contrast pictures of the internal anatomy. It can differentiate important elements of the atherosclerotic plaque with adequate reliability compared to the gold standard that was histopathological findings [20]. Magnetic resonance angiography (MRA) imaging is utilized to reduce the static tissue around the vessel, allowing for a more relevant picture of blood motion. Phase-contrast (PC) provides well-contrasted images by comparing the diastolic and systolic velocity waveforms, making it useful in localizing injuries. A similar, highly reliable noninvasive method that uses two images with and without contrast is the time of flight (TOF) MRA [42]. Computed tomography angiography (CTA) provides a highly accurate classification of the degree of CS with lower contrast use than standard angiography. Constant progress is being made to enhance the images and semi-automated algorithms, allowing for better diagnosis. DUS appears to be highly useful due to diverse factors. It offers realistic greyscale images that are beneficial for initial assessment; the addition of color and Doppler enhances the visualization of blood flow, making it a significantly important basic evaluation approach [43]. Although there are many possible diagnostic processes and options, DUS has been commonly used as a primary procedure due to its multiple immediate benefits and characteristics, including easy attainability, reliable images, and cost-effectiveness. CTA and MRA are usually the second and third most frequent diagnostic approaches in symptomatic patients [43]. Other alternatives like contrast-enhanced ultrasound (CEUS) have shown competitive results in the identification of vulnerable plaques with a sensitivity of 91% (95% CI: 84%, 95%), in some cases comparable to high-resolution MRI (HR-MRI) [44]. Other information has demonstrated that CTA and MRA showed increased accuracy than DUS when measuring CS of 70-99% of the stenotic artery (third-degree CS). However, highly specialized instruments and contrast-dependent studies can be difficult to access globally. Therefore, proper use of DUS has continued to be an excellent course of action as it has evidence that it can provide very similar results comparable to CTA when diagnosing second and third-degree CS [45]. Limitations of using DUS as the only diagnostic indicator should be cautiously considered. There is still plenty of progress and refinement being made in diagnostic techniques to continuously improve precision in diagnosis [46]. Development and research into innovative modalities utilizing different mechanisms to minimize radiation exposure and contrast administration and maximize diagnostic accuracy are underway. Some examples include optical coherence tomography (OCT), photoacoustic tomography (PAT) or thermoacoustic tomography (TAT), infrared thermography (IR), and video motion analysis (VMA). However, these diagnostic modalities are still in the developmental stage and are yet to be implemented in clinical practice [20, 47].

Management of carotid stenosis

Lifestyle Modifications

Diet: The Dietary Approaches to Stop Hypertension (DASH) diet, which is high in fruits, vegetables, and low-fat dairy and low in saturated fat and cholesterol, has been endorsed by the American Heart Association to enhance the cardiovascular health of the population [48]. The Mediterranean diet, which emphasizes fruits, vegetables, fish, and whole grains while allowing for the inclusion of low-fat dairy products, olive oil, and moderate alcohol use, has been shown to reduce stroke risk and CVD [49, 50]. Adopting a Mediterranean diet was associated with a moderate but statistically significant drop in blood pressure, with greater reductions seen in those with higher SBP at baseline and those who continued to be monitored for longer, depicted by a meta-analysis [51].

Exercise: At least 150-300 minutes per week of moderate-to-vigorous intensity aerobic physical exercise or an equal mix. Moreover, two days each week of muscle-strengthening activity [52]. In a multivariate study of individuals with severe cerebral atherosclerotic stenosis, physical inactivity increased the risk of recurrent stroke, heart attack, or vascular death by fivefold [53].

Smoking: Smoking is linked to first-time stroke and CVD; hence smoking cessation is emphasized to avoid future vascular occurrences [54]. To bring behavioral change, the 5As model is used for patient counseling (motivational interviewing), i.e., Ask: inquire about and document tobacco use; Advice: urge them to quit with clear and personalized language; Assess the patient’s willingness to quit; Assist: provide resources to help quit like cognitive behavioral therapy, nicotine replacement therapy (gum, patches, sprays, injections), pharmacotherapy (varenicline, bupropion), and support groups; and Arrange: schedule regular follow-ups in person or by telephone [55].

Medical management

Medical Management of Asymptomatic Patients

Antiplatelet use is controversial in asymptomatic individuals due to concerns that incorrect therapy may increase the risk of severe bleeding without lowering stroke risk. The odds of the benefits and risks of antiplatelet therapy in ACS should be analyzed. Patients with high-risk plaque are to be prescribed antiplatelet therapy [23]. However, a systematic review deduced that low-dose aspirin could not decrease vascular events but could mitigate the progression of CS [56]. Additionally, in a randomized case-control trial with 325 mg aspirin or placebo, conducted on asymptomatic patients with an audible cervical bruit with >50% stenosed ICA, no ischemic incidents or death occurred after a median of 2.3 years [57, 58]. Meta-analysis of trials of primary prevention aspirin allocation produced a 12% relative risk reduction (RRR) in major vascular events, partly because the net effect on stroke was not significant (0.20% vs. 0.21% per year, p = 0.4; hemorrhagic stroke 0.04% vs. 0.03%, p = 0.05%; other strokes, 0.16% vs. 0.018% per year, p = 0.08). Aspirin monotherapy is the first-line antiplatelet medication in asymptomatic patients, with clopidogrel reserved for aspirin-intolerant patients [58, 59]. A study indicated that early CEA reduced the risk of fatal and nonfatal strokes by less than half the risk difference in trials from 20 years earlier and was no longer statistically significant when non-stroke fatalities were considered; hence, medical therapy may be appropriate given the non-negligible 30-day perioperative hazards and stroke preventive improvements (Table 1) [60]. CREST-2 is conducting a trial on CEA/CAS in comparison to medical management alone in asymptomatic patients, which has yet to be concluded [61].

**Table 1:** Medical vs interventional therapy cohort study. CEA: Carotid endarterectomy.

Cohorts representing therapy type	Complications	Outcome	Confidence interval
CEA vs. medical therapy [60]	Fatal and non-fatal strokes Non-stroke fatalities	5.6% vs 7.8% with a risk difference of -2.3% Risk difference of -0.8%	95% CI: -4.0 to -0.3% 95% CI: -2.1 to 0.5%

Treatment of high blood pressure is related to a decrease and regression of CS; however, the benefit of antihypertensive medication on stroke prevention in individuals with ACS has not been fully examined [54]. Enalapril-folic acid reduced stroke risk significantly compared to enalapril alone in a Chinese trial [62]. The European Lacidipine Study on Atherosclerosis found that lacidipine (a calcium channel blocker [CCB]) reduced carotid intima-media thickness (CIMT) progression and atherosclerotic plaques more than atenolol, despite lesser BP decreases, suggesting an independent anti-atherosclerotic activity [59, 63]. Similar results have been reported with angiotensin-converting enzyme (ACE) inhibitors; however, CCBs diminish CIMT progression more than diuretics, beta-blockers, or ACE inhibitors [58, 64]. Antihypertensives were associated with a decreased incidence of ipsilateral stroke or transient ischemic attack (p = 0.01) and mortality from stroke or CVD (p = 0.0001). Blood pressure was substantially related to ipsilateral stroke or TIA and any stroke or CVD mortality. Blood pressure control mostly mediated the association between antihypertensives and ipsilateral stroke or TIA [65]. A review of 18 RCTs (56,934 participants) on statins in the primary prevention of cardiovascular disease found substantial decreases in all-cause mortality, fatal and non-fatal stroke, and revascularization operations [66]. The Asymptomatic Carotid Surgical Trial (ACST-1) showed a significantly lower 10-year incidence of stroke or death in participants receiving lipid-lowering medication and relatively lower perioperative risks. The absolute stroke risk rates in the patient who underwent immediate CEA on lipid-lowering medications were 0.7% vs. 1.3% per year [p<0·0001] as compared to 1·8% vs. 3·3% per year [p<0·0001] among those who were not [67]. Stringent glycemic control is needed in an ACS patient as the risk of stroke is doubled [58, 68]. In a trial of type II diabetes individuals who got risk factor counseling and adhered to a statin, antiplatelet, and antihypertensive medication (where appropriate), there was a 60% reduction in cardiovascular events (HR: 0.41%, 95% CI: 0.25-0.69, p = 0.001) and cardiovascular fatalities (HR: 0.43, 95% CI: 0.19-0.94, p = 0.04) [69].

Medical Management in Symptomatic Patients

Treatment with various antiplatelet medications, including aspirin, clopidogrel, and ticagrelor, has remained the mainstay of treatment for symptomatic CS, irrespective of extracranial or intracranial stenosis, to prevent complications like stroke and MI. Recent studies show that intensive medical therapy is the appropriate management of symptomatic intracranial stenosis, while extracranial stenosis of about 70% was found to have a better prognosis with revascularization. However, revascularization was found to be less beneficial with moderate stenosis, i.e., 50-69% [49]. Monotherapy with aspirin (low dose) or clopidogrel or the use of a combination of aspirin (low dose) and extended-release dipyridamole is preferred [70]. Two randomized control trials, aspirin-extended-release dipyridamole (ASA-ERDP) vs. clopidogrel and aspirin plus clopidogrel vs. aspirin plus dipyridamole, showed equal efficacy in reducing embolization and secondary stroke prevention (Table 2) [71, 72].

**Table 2:** RCTs on anti-platelets use in symptomatic carotid stenosis patients. ASA-ERDP: Aspirin-extended release dipyridamole; RCTs: Randomized controlled trials.

Study	Year	Drugs	Complication prevention	Outcome
King A et al. [71]	2011	Aspirin plus dipyridamole vs aspirin plus clopidogrel	Embolization/Stroke	Aspirin + Dipyridamole vs. Aspirin + Clopidogrel (75.5% vs. 77.5%; P=0.77)
Sacco RL et al. [72]	2008	ASA-ERDP vs. clopidogrel	Recurrent stroke, MI and vascular death. Major haemorrhage	2 treatment groups were the same (13.1%; 4.1% vs. 3.6%; HR 1.15, 95% CI: 1.00-1.32)

In individuals with acutely symptomatic CS, short-term preoperative anticoagulation is safe and beneficial in preventing recurrent cerebral ischemic episodes while awaiting CEA, compared to antiplatelet medications alone (Table 3) [73].

**Table 3:** Antiplatelet vs. anticoagulants in symptomatic carotid stenosis patients. TIA: Transient ischemic attack.

Cohort groups	Risk prevention	Outcome
Anticoagulants vs. antiplatelet [73]	Recurrent stroke/TIA	3.8 vs. 10.9%, p=0.006
	Intracranial haemorrhage	Patient vs. 0 patient

A significant risk reduction ratio (RRR) was noted with antihypertensive therapy, as suggested by a meta-analysis of 13 blood pressure trials [74]. Preoperatively, patients on ≥2 antihypertensive drugs can lead to post-op hypotension [75]. A clinicopathological investigation reveals that statin treatment stabilizes the atherosclerotic plaques due to a decrease in intra-plaque microvessels, lowering VEGF and decreasing inflammation, in the carotid artery, in addition to a reduction in the volume of infarcts in symptomatic patients undergoing CEA [76, 77]. This stability of the plaques may have a pleiotropic impact in addition to the lipid-lowering action of statins [78]. The management of patients with diabetes is the same as for asymptomatic patients.

Perioperative Medical Management

Patients are recommended to take aspirin before CEA [79]. Patients who did not receive perioperative antiplatelet medication had a significantly higher combined stroke and mortality rate (risk ratio [RR], 1.21; 95% CI: 1.04-1.42) than those who received monotherapy (single antiplatelet therapy [sAPT]). However, dual antiplatelet therapy (dAPT) was linked with a lower risk of death (RR: 0.67; 95% CI: 0.51-0.88) but a considerably increased risk of subsequent bleeding necessitating reoperation (RR: 2.16; 95% CI: 1.50-2.50) [80]. In a nutshell, dAPT has a higher risk for bleeding and reoperation than sAPT [80, 81]. In contrast, a study concluded that sAPT vs. dAPT did not determine significant differences in perioperative bleeding [82]. Beta-blockers may cause intraoperative hemodynamic depression, especially in individuals with low baseline heart rates [83]. Prior to CEA and CAS, statin treatment significantly reduced the 30-day risk of stroke and perioperative mortality (Table 4) [84-86].

**Table 4:** Perioperative medical management. OR: Odds ratio; MI: Myocardial infarction.

Study	Year	Risk/complications	Outcome of pre-treatment with and without statins
Groschel K et al. [84]	2004	Stroke/Perioperative death/MI	4% vs. 15% (OR: 0.23, 95% CI: 0.05-0.99, p=0.049)
Verzini F et al. [85]	2011		OR: 0.33, 95% CI: 0.13-0.80, p=0.016)
Reiff T et al. [86]	2014		6.8% vs. 13.9% (OR: 0.31, 95% CI: 0.3-0.71, p=0.006)

Postprocedure Medical Management

Patients who use dAPT were observed to be more adherent than those who use sAPT [87]. While receiving dAPT, the rate of extracranial hemorrhage was nearly six times higher (6.50% per patient-month vs. 1.16% per patient-month, p = 0.001%), and the rate of intracranial hemorrhage showed a trend but was not statistically significant. Extracranial hemorrhagic episodes 30 days after CAS were substantially more likely to occur (p = 0.028). The possible advantage of extending dAPT with respect to ischemic consequences must be weighed against the increased risk of predominantly extracranial hemorrhagic complications [88]. Post-CEA or CAS, antiplatelet therapies have shown an increased risk of stroke with <180 days of prescribed post-CAS P2Y12-inhibition (HR = 1.421, 95% CI: 1.038-1.946; 90-179 prescribed days: HR = 1.484, 95% CI: 1.045-2.106). Moreover, the incidence of hemorrhagic complications was also higher (1.16% per person-month vs. 0.49% per person-month after discontinuation, p = 0.001) [88]. P2Y12 inhibitors can therefore be administered to aspirin-allergic or intolerant patients alone [23]. When protamine is administered, aspirin-ticagrelor is associated with a reduced risk of stroke/death (1.5% vs. 3.9%; RR: 0.38) and bleeding (3.0% vs. 2.6%; RR: 1.1) complications following CAS compared to aspirin-clopidogrel. However, when protamine is not administered, aspirin-ticagrelor is associated with a higher risk of stroke/death (4.1% vs. 2.6%; RR: 1.5) and bleeding (5.8% vs. 2.8%; RR: 2.0) [89]. Using aspirin with clopidogrel or ticagrelor for a brief period of 1-3 months, followed by aspirin alone, can prevent strokes [90, 91, 92]. Beta-blockers prevent post-CEA hypertension and stabilize postoperative peak systolic blood pressure three days following conventional endarterectomy [83]. Statins are continued after the interventional procedures to avoid further risk of complications. Studies show that the risk of cardiovascular complications, including myocardial infarction, congestive cardiac failure, and dysrhythmia, is increased postprocedure [93, 94]. Statin use reduces the risk of cardiovascular complications and provides long-term protection from stroke. High-dose statins did not show statistical significance compared to normal-dose statins [95].

Surgical and Endovascular Management of Carotid Stenosis

**Figure 2:**
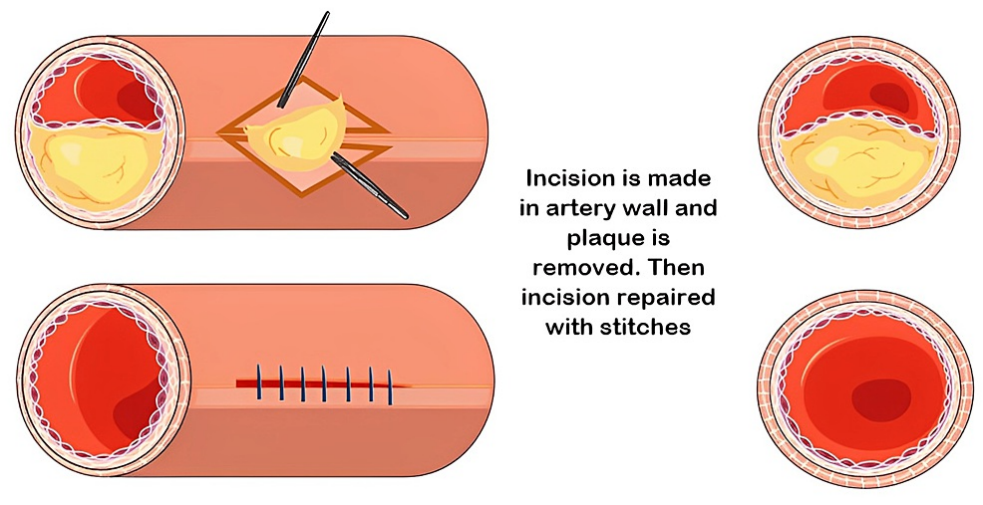
Carotid endarterectomy. Source: Parts of the figure were drawn by using pictures from Servier Medical Art. Servier Medical Art by Servier is licensed under a Creative Commons Attribution 3.0 Unported License (https://creativecommons.org/licenses/by/3.0/).

**Figure 3:**
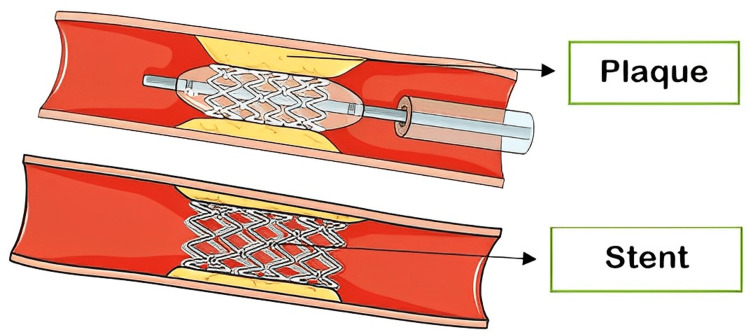
Carotid stenting. Source: Parts of the figure were drawn by using pictures from Servier Medical Art. Servier Medical Art by Servier is licensed under a Creative Commons Attribution 3.0 Unported License (https://creativecommons.org/licenses/by/3.0/).

CS leading to permanent or transient neurological or visual symptoms in the preceding six months is categorized as symptomatic, and it is essential to evaluate and treat these patients [96] immediately. As per the clinical situation, carotid intervention, CEA, and CAS are mostly advised in symptomatic patients with more than 70% stenosis, as well as in certain asymptomatic low-risk patients and symptomatic patients with stenosis of 50-69% (Figures 2-3) [97]. CEA became the conventionally accepted standard of care for severe (>70% stenosis) symptomatic CS after the North American Carotid Endarterectomy Trial (NASCET) was published. CEA for asymptomatic CS also gained popularity after two randomized clinical trials showed an absolute risk reduction of 5-6% compared to non-surgical treatment [37]. 

In CEA classical approach, the external, internal, and common carotid arteries are cross-clamped so that the carotid bifurcation is isolated from the circulation. The artery is opened, and the plaque is removed. Most often, this is done through a longitudinal incision, and the artery is patched upon closure as this reduces the incidence of restenosis. The operation of eversion CEA, in which the internal carotid artery is transected and turned inside out to remove the plaque, is used by some surgeons. Whichever technique is used, care must be taken to remove all of the debris from the intimal surface of the artery to prevent postoperative emboli from entering the circulation [98].

The use of local anesthesia is common during CAS. It entails inserting a wire beyond the stenotic lesion and either using an umbrella-like filter to catch any atheroma fragments that become loose during the stent placement or inflating balloons in the external and common carotid arteries to encourage reverse flow down the internal carotid, hence preventing atheromatous emboli from being carried up into the intracerebral circulation [99]. According to the guidelines of the Society of Vascular Surgery, in symptomatic patients with >50% stenosis, carotid revascularization should be carried out as soon as the patient is neurologically stable after 48 hours, but most likely before 14 days following the onset of symptoms. They advised CEA over carotid stenting in patients receiving revascularization during the first 14 days of the start of symptoms. To prevent stroke and mortality over the long term in low surgical-risk individuals with asymptomatic carotid bifurcation atherosclerosis and stenosis of >70% (confirmed by validated DUS or CTA/angiography), it advised combining CEA with the best medical therapy (BMT) [100]. In order to compare the relative effectiveness of CAS and CEA for the treatment of symptomatic CS, some researchers aggregated the data collected at the individual patient level from the four major randomized controlled studies (Endarterectomy versus Angioplasty in Patients with Symptomatic Severe Carotid Stenosis trial, Stent-Protected Percutaneous Angioplasty of the Carotid Artery versus Endarterectomy trial, International Carotid Stenting Study, and Carotid Revascularization Endarterectomy versus Stenting Trial). A total of 4754 (99.6%) of the 4775 patients who were randomly assigned to the four included studies were followed up for a maximum of 12 years. Those assigned CEA experienced 129 periprocedural and 55 postprocedure outcome events like different types of stroke and death, compared to 206 and 57 for patients assigned who underwent CAS. Results following both procedures were comparable in the postoperative phase, with the annual rates of ipsilateral stroke per person-year being 0·60% (95% CI: 0·46-0·79) for CEA and 0·64% (0·49-0·83) for CAS, respectively. The comparability of the postprocedure rates suggested that advances in the periprocedural safety of CAS could yield similar outcomes, even while long-term outcomes (periprocedural and postprocedure concerns combined) continue to favor CEA [101]. The majority of this increased risk is related to a rise in mild, non-disabling strokes in adults over the age of 70 [102]. High-risk characteristics for CEA should be examined because CAS provides an option for patients who are ineligible for CEA. Non-vascular characteristics of high-risk CEA include high cervical lesions and challenging surgical fields. Typically, CEA is considered inappropriate for carotid lesions above the C2 level. High carotid exposure can raise the risk of cranial nerve damage, particularly the vagus nerve. The presence of tracheal stroma, prior neck surgery or radiation, and fibrotic or scarred alterations to the surrounding connective tissues can make surgical dissection more difficult during CEA. Having a short neck due to obesity or having previously undergone cervical fusion can also make CEA more difficult. Patients with a contralateral vocal cord injury or laryngeal nerve palsy are viewed as being ineligible for CEA because of the apparent danger of cranial nerve harm [58, 101, 103]. In patients who underwent CEA, restenosis was more likely in smokers than in non-smokers, but no difference was seen in patients who received CAS [104]. Regardless of treatment, patients who suffered restenosis were more likely to be female, hypertensive, diabetic, and had a deranged lipid profile than those who did not. It was concluded in a 2016 review that intensive medical therapy would be a superior course of treatment for the majority of ACS patients than either stenting or endarterectomy. Transcranial Doppler ultrasound (TCD) embolus detection or other emerging techniques can be used to find the selected few (10%) who potentially benefit from intervention [105]. Trans-carotid artery revascularization is one of the developments in the invasive management of carotid disease and has already received limited approval from the US FDA. Due to enrollment, regulatory, and financing obstacles, it may be difficult to demonstrate the safety and effectiveness of trans-carotid artery revascularization compared to CEA, CAS, or BMT alone [106]. Major surgical intervention trials are summarized in Table 5.

**Table 5:** Summary of major trials on interventional procedures for carotid stenosis. NASCET: North American Symptomatic Carotid Endarterectomy Trial; ACST: Asymptomatic Carotid Surgery Trial; CREST: Carotid Revascularization Endarterectomy Versus Stent Trial; SPACE: Stent-Supported Percutaneous Angioplasty of the Carotid Artery vs. Endarterectomy Trial; CEA: Carotid endarterectomy; MI: Myocardial infarction; CAS: Carotid artery stenosis; BMT: Best medical therapy.

Study	Duration of Study	Sample Size n	Findings	Outcome
NASCET [107, 108]	1987-1999	1415	Perioperative stroke and death calculated among patients who underwent CEA.	The overall rate of perioperative stroke and death was 6.5%, while the rate of permanently disabling stroke and death was only 2.0%.
ACST-1 [67]	1993-2003	3120	Perioperative risk of stroke and death among asymptomatic patient who underwent CEA vs who deferred the procedure.	When combining perioperative events and strokes, the net risks were 6.9% versus 10.9% at 5 years (a gain of 4.1%, 2.0-6.2), and 13.4% versus 17.9% at 10 years (a gain of 4.6%, 1.2-7.9).
CREST [109]	2000-2010	2502	The 4-year rate of stroke, death, or MI was evaluated among asymptomatic or symptomatic patients who underwent either CEA or CAS.	The 4-year rate of stroke or death was 6.4% with stenting and 4.7% with endarterectomy (HR: 1.50; P=0.03). Among symptomatic patients, the rates were 8.0% and 6.4% (HR: 1.37; P=0.14), and among asymptomatic patients, the rates were 4.5% and 2.7% (HR: 1.86; P=0.07), respectively. At the 10-year follow-up, post-procedural stroke occurred in 6.9% (95% CI: 4.4-9.7) of patients in the stenting group and 5.6% (95% CI: 3.7-7.6) of those in the endarterectomy group. The difference was not statistically significant (HR: 0.99; 95% CI: 0.64-1.52).
SPACE-1 [110]	2001-2006	1214	The 2-year rate of stroke and restenosis was compared between patients who underwent carotid angioplasty with a stent and those who underwent carotid endarterectomy.	Restenosis occurred more frequently in the stenting group than in the endarterectomy group, both in the intention-to-treat and per-protocol analyses. The life-table estimates were 10.7% versus 4.6% (p=0.0009) and 11.1% versus 4.6% (p=0.0007), respectively. After a 2-year follow-up, the rate of recurrent ipsilateral ischemic strokes reported in the SPACE trial was similar for both treatment groups.
SPACE -2 [111]	2009-2019	513	Post-procedural 1-month and 5-year risk of stroke was calculated among patients who received CEA plus BMT, CAS plus BMT, or BMT alone.	The cumulative incidence of any stroke or death from any cause within 30 days, or any ipsilateral ischemic stroke within 5 years (the primary efficacy endpoint), was 2.5% (95% CI: 1.0-5.8) with CEA plus BMT, 4.4% (2.2-8.6) with CAS plus BMT, and 3.1% (1.0-9.4) with BMT alone. Neither CEA plus BMT nor CAS plus BMT was found to be superior to BMT alone.
ACST-2 [112]	2008-2020	3625	The 5-year risk of stroke was calculated among asymptomatic patients with severe stenosis who underwent either CAS or CEA.	The 5-year non-procedural stroke rates were identical in each group for fatal or disabling stroke, at 2.5%. For any stroke, the rate was 5.3% with CAS versus 4.5% with CEA (rate ratio [RR] 1.16, 95% CI: 0.861.57; p = 0.33).

Complications

General Complications of Carotid Stenosis

If left untreated, there are several potential complications associated with CS, such as embolism, aneurysm development, and even an increased risk of myocardial infarction [113, 114]. An embolism results from a thrombus or plaque fragment becoming dislodged and migrating to the cerebral circulation, obstructing a smaller artery and ultimately leading to either a transient ischemic attack (TIA) or a stroke. It is noteworthy that up to 15% of ischemic strokes have been attributed to pre-existing ACS exceeding 50% obstruction [58, 115]. Furthermore, CS can lead to the weakening of artery walls, which in turn can result in the development of an aneurysm. However, this is a rare complication, constituting less than 1% of all arterial aneurysms [116]. It is important to note that most of these aneurysms are pseudoaneurysms (PA), while less than 10% are true aneurysms [117, 118]. As atherosclerosis is a vascular disorder characterized by systemic inflammation, research indicates that individuals with CS are at an elevated risk for various types of atherosclerotic cardiovascular events, particularly those related to the coronary arteries [119]. The incidence of significant carotid artery disease in patients referred for symptomatic coronary artery disease (CAD) has been reported to vary from 7% to 36% [120]. Additionally, a positive correlation has been observed between the degree of stenosis in the internal carotid artery and the severity of CAD [121]. The risk of these complications associated with CS can be reduced through early diagnosis and treatment, including medical management and/or surgical intervention. Regular monitoring and management of risk factors, such as high blood pressure, high cholesterol, and diabetes, can also help to reduce the risk of complications [122].

Complications of Surgical Management

CS surgical treatments, including CEA and CAS, are effective in improving blood flow to the brain by removing or opening the blockage in the carotid arteries. However, these procedures also carry potential risks and complications. The most severe complication of both CEA and CAS is stroke, which can occur due to dislodging of plaque or a blood clot during the procedure. CAS is associated with an increased risk of perioperative distal microembolization, which may arise from plaque remodeling soon after stent implantation. While most microembolization may be asymptomatic, they may also give rise to neurological events and cognitive impairment [123]. Post-endarterectomy pseudoaneurysm (PEPA) or pseudoaneurysm formation following CEA is an infrequent occurrence, accounting only for 0.3%-0.6% of all procedures [124, 125]. PAs after CEA is not limited to the immediate postoperative period since evidence indicates that their development could take place several years after the initial CEA operation [126]. Bleeding is another potential complication that can occur during or after the procedure. Infection is also a possible risk, occurring at the incision site or within the carotid artery. In rare cases, CEA and CAS can trigger a heart attack, particularly in people with underlying heart disease. Additionally, nerve damage can occur during CEA, leading to temporary or permanent weakness, numbness in the face, or difficulty swallowing. Restenosis might occur as a long-term complication that can occur due to the recurrence of blockage in the carotid artery after CEA and CAS. There has been inconsistency in the research regarding the durability of carotid artery patency over time following each respective procedure [127-129]. However, in 2018, a randomized controlled trial found that moderate (≥50%) restenosis occurred more frequently in stenting than in endarterectomy, with resulting cumulative five-year risks of 40.7% versus 29.6%, respectively [130]. 

Complications of Medical Management

CS medical treatment aims to manage the symptoms and slow down the progression of the condition by controlling risk factors such as high blood pressure, high cholesterol, and diabetes. The most common medical treatments for CS are antiplatelet medications (such as aspirin) and statins. While these treatments are generally safe and well-tolerated, they can still have potential complications, including bleeding, allergic reactions, weakness and myositis, and even liver damage in patients with pre-existing liver disease [116]. The comprehensive evaluation of the potential complications associated with medical treatment in patients with ACS is of paramount importance. Especially the risk of stroke, given that prior research has demonstrated that even a 10% increase in stenosis severity can lead to doubling stroke risk [131, 132]. Patients who have experienced silent infarction, contralateral transient cerebral ischemic attack, or stroke may face a threefold increase in their risk of stroke [133]. Additionally, the risk may be amplified by factors such as impaired cerebrovascular reserve or spontaneous microembolization, which could elevate the risk by six times or more [134].

## Conclusions

Our review of the current literature on CS has shed light on the diagnostic and management strategies and correlated complications. CS is the fragment of the soaring global cardiovascular burden, which leads to debilitating neurological symptoms and stroke. Efficient diagnosis and classification of CS are crucial for proper management, and various diagnostic methods, including DSA, MRI, MRA, CTA, and DUS, are available. DUS is the widely accepted primary modality due to its attainability, immediate, reliable images, and cost-effectiveness. For asymptomatic and mild or moderate symptomatic CS, antiplatelet and lipid-lowering medications are the optimal medical treatments to prevent complications like stroke and myocardial infarction, along with adequate control of comorbidities like diabetes mellitus and hypertension. Carotid intervention is recommended for symptomatic patients, most commonly with severe stenosis. While CEA and CAS are the two primary surgical approaches used to treat CS, they carry potential risks and complications such as stroke, bleeding, infection, and nerve damage. Therefore, it is essential to identify predictive factors for adverse outcomes in individual patients to minimize the risk of complications. Since CS is a multifactorial disease, a standardized protocol may not be the most appropriate approach for treatment. Instead, personalized treatment plans based on individual patient characteristics and risk factors may be more effective. In conclusion, while current medical and surgical treatments for CS have shown a reduced risk of stroke and other complications, there is still a dire need for further research and development of preventive strategies to minimize the incidence and mitigate the complications of the disease.

## References

[1] Teng Z, Sadat U, Brown AJ, Gillard JH (2014). Plaque hemorrhage in carotid artery disease: pathogenesis, clinical and biomechanical considerations. J Biomech.

[2] Bir SC, Kelley RE (2022). Carotid atherosclerotic disease: a systematic review of pathogenesis and management. Brain Circ.

[3] Sperber GH (2006). Clinically oriented anatomy. J Anat.

[4] Standring S (2020). Gray’s Anatomy: The Anatomical Basis of Clinical Practice. https://www.scirp.org/(S(351jmbntvnsjt1aadkposzje))/reference/ReferencesPapers.aspx?ReferenceID=1830308.

[5] Sethi D, Gofur EM, Munakomi S (2023). Anatomy, Head and Neck: Carotid Arteries. https://www.ncbi.nlm.nih.gov/books/NBK545238/.

[6] Bouthillier A, van Loveren HR, Keller JT (1996). Segments of the internal carotid artery: a new classification. Neurosurgery.

[7] Kumar P, Prabhakar NR (2012). Peripheral chemoreceptors: function and plasticity of the carotid body. Compr Physiol.

[8] Butt N, Baek WK, Lachkar S (2019). The carotid body and associated tumors: updated review with clinical/surgical significance. Br J Neurosurg.

[9] West CT, Brassett C, Gaunt ME (2018). Variations in carotid sinus anatomy and their relevance to carotid interventions. Folia Morphol (Warsz).

[10] Guirguis-Blake JM, Webber EM, Coppola EL (2021). Screening for asymptomatic carotid artery stenosis in the general population: updated evidence report and systematic review for the US Preventive Services Task Force. JAMA.

[11] Prasad K (2015). Pathophysiology and medical treatment of carotid artery stenosis. Int J Angiol.

[12] Autret A, Pourcelot L, Saudeau D, Marchal C, Bertrand P, de Boisvilliers S (1987). Stroke risk in patients with carotid stenosis. Lancet.

[13] Doonan RJ, Abdullah A, Steinmetz-Wood S (2019). Carotid endarterectomy outcomes in the elderly: a Canadian institutional experience. Ann Vasc Surg.

[14] Morales MM, Anacleto A, Filho CM, Ledesma S, Aldrovani M, Wolosker N (2019). Peak systolic velocity for calcified plaques fails to estimate carotid stenosis degree. Ann Vasc Surg.

[15] Johansson A, Acosta S (2020). Diet and lifestyle as risk factors for carotid artery disease: a prospective cohort study. Cerebrovasc Dis.

[16] Cheng SF, Brown MM, Simister RJ, Richards T (2019). Contemporary prevalence of carotid stenosis in patients presenting with ischaemic stroke. Br J Surg.

[17] Rockman CB, Hoang H, Guo Y (2013). The prevalence of carotid artery stenosis varies significantly by race. J Vasc Surg.

[18] Kim HW, Regenhardt RW, D'Amato SA (2022). Asymptomatic carotid artery stenosis: a summary of current state of evidence for revascularization and emerging high-risk features. J Neurointerv Surg.

[19] Giannopoulos A, Kakkos S, Griffin MB, Geroulakos G, Tsalikakis D, Nicolaides A (2019). Mortality risk stratification in patients with asymptomatic carotid stenosis. Vasc Invest Ther.

[20] Saxena A, Ng EY, Lim ST (2019). Imaging modalities to diagnose carotid artery stenosis: progress and prospect. Biomed Eng Online.

[21] Abbott AL, Paraskevas KI, Kakkos SK (2015). Systematic review of guidelines for the management of asymptomatic and symptomatic carotid stenosis. Stroke.

[22] Rothwell PM (2006). Medical and surgical management of symptomatic carotid stenosis. Int J Stroke.

[23] Hackam DG (2021). Optimal medical management of asymptomatic carotid stenosis. Stroke.

[24] Song P, Fang Z, Wang H (2020). Global and regional prevalence, burden, and risk factors for carotid atherosclerosis: a systematic review, meta-analysis, and modelling study. Lancet Glob Health.

[25] Woo SY, Joh JH, Han SA, Park HC (2017). Prevalence and risk factors for atherosclerotic carotid stenosis and plaque: a population-based screening study. Medicine (Baltimore).

[26] Hong YM (2010). Atherosclerotic cardiovascular disease beginning in childhood. Korean Circ J.

[27] Peng M, Li X, Liu Y, Zou M, Xia Y, Xu G (2020). Dietary glycemic index and glycemic load in relation to atherosclerotic stenosis of carotid and cardiovascular risk factors in ischemic stroke patients. J Atheroscler Thromb.

[28] Zanoli L, Mikhailidis DP (2021). Narrative review of carotid disease and the kidney. Ann Transl Med.

[29] Khaleghi M, Isseh IN, Jouni H, Sohn S, Bailey KR, Kullo IJ (2014). Family history as a risk factor for carotid artery stenosis. Stroke.

[30] Meng R, Mi X, Sun D (2019). Risk factors for recurrent carotid-artery stenosis following stenting treatment. Med Sci Monit.

[31] Buonacera A, Stancanelli B, Malatino L (2019). Stroke and hypertension: an appraisal from pathophysiology to clinical practice. Curr Vasc Pharmacol.

[32] Amarenco P, Goldstein LB, Callahan A 3rd (2009). Baseline blood pressure, low- and high-density lipoproteins, and triglycerides and the risk of vascular events in the Stroke Prevention by Aggressive Reduction in Cholesterol Levels (SPARCL) trial. Atherosclerosis.

[33] Cho HM, Kang DR, Kim HC, Oh SM, Kim BK, Suh I (2015). Association between fibrinogen and carotid atherosclerosis according to smoking status in a Korean male population. Yonsei Med J.

[34] Ehrhardt J, Schwab M, Finn S, Guenther A, Schultze T, Witte OW, Rupprecht S (2015). Sleep apnea and asymptomatic carotid stenosis: a complex interaction. Chest.

[35] Deeb R, Smeds MR, Bath J (2019). Snoring and carotid artery disease: a new risk factor emerges. Laryngoscope.

[36] Gray VL, Desikan SK, Khan AA, Barth D, Sikdar S, Sorkin JD, Lal BK (2021). Revascularization for asymptomatic carotid artery stenosis improves balance and mobility. J Vasc Surg.

[37] Heck D, Jost A (2021). Carotid stenosis, stroke, and carotid artery revascularization. Prog Cardiovasc Dis.

[38] McColgan P, Bentley P, McCarron M, Sharma P (2012). Evaluation of the clinical utility of a carotid bruit. QJM.

[39] Lal BK, Dux MC, Sikdar S, Goldstein C, Khan AA, Yokemick J, Zhao L (2017). Asymptomatic carotid stenosis is associated with cognitive impairment. J Vasc Surg.

[40] Del Brutto VJ, Gornik HL, Rundek T (2020). Why are we still debating criteria for carotid artery stenosis?. Ann Transl Med.

[41] Lalla R, Raghavan P, Chaturvedi S (2020). Trends and controversies in carotid artery stenosis treatment. F1000Res.

[42] Tapis P, El-Koussy M, Hewer E, Mono ML, Reinert M (2020). Plaque vulnerability in patients with high- and moderate-grade carotid stenosis - comparison of plaque features on MRI with histopathological findings. Swiss Med Wkly.

[43] Saba L, Mossa-Basha M, Abbott A (2021). Multinational survey of current practice from imaging to treatment of atherosclerotic carotid stenosis. Cerebrovasc Dis.

[44] Li Q, Cai M, Wang H, Chen L (2023). Diagnostic performance of contrast-enhanced ultrasound and high-resolution magnetic resonance imaging for carotid atherosclerotic plaques: a systematic review and meta-analysis. J Ultrasound Med.

[45] Rustempasic N, Gengo M (2019). Assesment of carotid stenosis with CT angiography and color Doppler ultrasonography. Med Arch.

[46] Cassola N, Baptista-Silva JC, Nakano LC (2022). Duplex ultrasound for diagnosing symptomatic carotid stenosis in the extracranial segments. Cochrane Database Syst Rev.

[47] Tsai CH, Huang CC, Hsiao HM (2022). Detection of carotid artery stenosis based on video motion analysis for fast screening. J Am Heart Assoc.

[48] Belanger MJ, Kovell LC, Turkson-Ocran RA (2023). Effects of the dietary approaches to stop hypertension diet on change in cardiac biomarkers over time: results from the DASH-Sodium trial. J Am Heart Assoc.

[49] Wabnitz AM, Turan TN (2017). Symptomatic carotid artery stenosis: surgery, stenting, or medical therapy?. Curr Treat Options Cardiovasc Med.

[50] Estruch R, Ros E, Salas-Salvadó J (2013). Primary prevention of cardiovascular disease with a Mediterranean diet. N Engl J Med.

[51] Filippou CD, Thomopoulos CG, Kouremeti MM, Sotiropoulou LI, Nihoyannopoulos PI, Tousoulis DM, Tsioufis CP (2021). Mediterranean diet and blood pressure reduction in adults with and without hypertension: a systematic review and meta-analysis of randomized controlled trials. Clin Nutr.

[52] Lane-Cordova AD, Jerome GJ, Paluch AE (2022). Supporting physical activity in patients and populations during life events and transitions: a scientific statement from the American Heart Association. Circulation.

[53] Turan TN, Nizam A, Lynn MJ (2017). Relationship between risk factor control and vascular events in the SAMMPRIS trial. Neurology.

[54] Sutton-Tyrrell K, Wolfson SK Jr, Kuller LH (1994). Blood pressure treatment slows the progression of carotid stenosis in patients with isolated systolic hypertension. Stroke.

[55] (2023). Five Major Steps to Intervention (The "5 A's"). https://www.ahrq.gov/prevention/guidelines/tobacco/5steps.html#:~:text=Successful%20intervention%20begins%20with%20identifying,Assess%2C%20Assist%2C%20and%20Arrange.

[56] Hu X, Hu Y, Sun X, Li Y, Zhu Y (2022). Effect of aspirin in patients with established asymptomatic carotid atherosclerosis: a systematic review and meta-analysis. Front Pharmacol.

[57] Côté R, Battista RN, Abrahamowicz M, Langlois Y, Bourque F, Mackey A (1995). Lack of effect of aspirin in asymptomatic patients with carotid bruits and substantial carotid narrowing. The Asymptomatic Cervical Bruit Study Group. Ann Intern Med.

[58] Naylor AR, Ricco JB, de Borst GJ (2018). Editor's Choice - management of atherosclerotic carotid and vertebral artery disease: 2017 Clinical Practice Guidelines of the European Society for Vascular Surgery (ESVS). Eur J Vasc Endovasc Surg.

[59] Baigent C, Blackwell L, Collins R (2009). Aspirin in the primary and secondary prevention of vascular disease: collaborative meta-analysis of individual participant data from randomised trials. Lancet.

[60] Keyhani S, Cheng EM, Hoggatt KJ (2020). Comparative effectiveness of carotid endarterectomy vs initial medical therapy in patients with asymptomatic carotid stenosis. JAMA Neurol.

[61] Lal BK, Meschia JF, Brott TG (2017). Clinical need, design, and goals for the Carotid Revascularization and Medical Management for Asymptomatic Carotid Stenosis trial. Semin Vasc Surg.

[62] Huo Y, Li J, Qin X (2015). Efficacy of folic acid therapy in primary prevention of stroke among adults with hypertension in China: the CSPPT randomized clinical trial. JAMA.

[63] Zanchetti A, Bond MG, Hennig M (2002). Calcium antagonist lacidipine slows down progression of asymptomatic carotid atherosclerosis: principal results of the European Lacidipine Study on Atherosclerosis (ELSA), a randomized, double-blind, long-term trial. Circulation.

[64] Wang JG, Staessen JA, Li Y (2006). Carotid intima-media thickness and antihypertensive treatment: a meta-analysis of randomized controlled trials. Stroke.

[65] King A, Shipley M, Markus H (2013). The effect of medical treatments on stroke risk in asymptomatic carotid stenosis. Stroke.

[66] Taylor F, Huffman MD, Macedo AF (2013). Statins for the primary prevention of cardiovascular disease. Cochrane Database Syst Rev.

[67] Halliday A, Harrison M, Hayter E (2010). 10-year stroke prevention after successful carotid endarterectomy for asymptomatic stenosis (ACST-1): a multicentre randomised trial. Lancet.

[68] Banerjee C, Moon YP, Paik MC (2012). Duration of diabetes and risk of ischemic stroke: the Northern Manhattan Study. Stroke.

[69] Gaede P, Lund-Andersen H, Parving HH, Pedersen O (2008). Effect of a multifactorial intervention on mortality in type 2 diabetes. N Engl J Med.

[70] Brott TG, Halperin JL, Abbara S (2011). 2011 ASA/ACCF/AHA/AANN/AANS/ACR/ASNR/CNS/SAIP/SCAI/SIR/SNIS/SVM/SVS guideline on the management of patients with extracranial carotid and vertebral artery disease. Stroke.

[71] King A, Bath PM, Markus HS (2011). Clopidogrel versus dipyridamole in addition to aspirin in reducing embolization detected with ambulatory transcranial Doppler: a randomized trial. Stroke.

[72] Sacco RL, Diener HC, Yusuf S (2008). Aspirin and extended-release dipyridamole versus clopidogrel for recurrent stroke. N Engl J Med.

[73] Martinez-Gutierrez JC, Roy AT, D'Amato S (2022). Preoperative antithrombotic treatment in acutely symptomatic carotid artery stenosis. J Stroke Cerebrovasc Dis.

[74] Law MR, Morris JK, Wald NJ (2009). Use of blood pressure lowering drugs in the prevention of cardiovascular disease: meta-analysis of 147 randomised trials in the context of expectations from prospective epidemiological studies. BMJ.

[75] Rubio G, Karwowski JK, DeAmorim H, Goldstein LJ, Bornak A (2019). Predicting factors associated with postoperative hypotension following carotid artery stenting. Ann Vasc Surg.

[76] Sahebkar A, Ponziani MC, Goitre I, Bo S (2015). Does statin therapy reduce plasma VEGF levels in humans? A systematic review and meta-analysis of randomized controlled trials. Metabolism.

[77] Silvestre JS, Mallat Z, Tedgui A, Lévy BI (2008). Post-ischaemic neovascularization and inflammation. Cardiovasc Res.

[78] Konishi T, Funayama N, Yamamoto T (2018). Stabilization of symptomatic carotid atherosclerotic plaques by statins: a clinico-pathological analysis. Heart Vessels.

[79] Schoenefeld E, Donas K, Radicke A, Osada N, Austermann M, Torsello G (2012). Perioperative use of aspirin for patients undergoing carotid endarterectomy. Vasa.

[80] Zimmermann A, Knappich C, Tsantilas P (2018). Different perioperative antiplatelet therapies for patients treated with carotid endarterectomy in routine practice. J Vasc Surg.

[81] Ku JC, Taslimi S, Zuccato J (2022). Editor's Choice - Peri-operative outcomes of carotid endarterectomy are not improved on dual antiplatelet therapy vs. aspirin monotherapy: a systematic review and meta-analysis. Eur J Vasc Endovasc Surg.

[82] Vezir Ö, Erturk E (2019). Comparison of preoperative single and dual antiplatelet therapy in bleeding after carotid endarterectomy. Koşuyolu Heart J.

[83] Teng L, Fang J, Zhang Y, Liu X, Qu C, Shen C (2021). Perioperative baseline β-blockers: An independent protective factor for post-carotid endarterectomy hypertension. Vascular.

[84] Gröschel K, Ernemann U, Schulz JB, Nägele T, Terborg C, Kastrup A (2006). Statin therapy at carotid angioplasty and stent placement: effect on procedure-related stroke, myocardial infarction, and death. Radiology.

[85] Verzini F, De Rango P, Parlani G (2011). Effects of statins on early and late results of carotid stenting. J Vasc Surg.

[86] Reiff T, Amiri H, Rohde S, Hacke W, Ringleb PA (2014). Statins reduce peri-procedural complications in carotid stenting. Eur J Vasc Endovasc Surg.

[87] Lee M, Ahmed ZV, Huang J (2022). Antiplatelet regimens following carotid artery revascularization. Am Heart J.

[88] Sussman ES, Jin M, Pendharkar AV, Pulli B, Feng A, Heit JJ, Telischak NA (2021). Dual antiplatelet therapy after carotid artery stenting: trends and outcomes in a large national database. J Neurointerv Surg.

[89] Marcaccio CL, Patel PB, Liang P (2022). Efficacy and safety of perioperative dual antiplatelet therapy with ticagrelor versus clopidogrel in carotid artery stenting. J Vasc Surg.

[90] Johnston SC, Easton JD, Farrant M (2018). Clopidogrel and aspirin in acute ischemic stroke and high-risk TIA. N Engl J Med.

[91] Johnston SC, Amarenco P, Denison H (2020). Ticagrelor and aspirin or aspirin alone in acute ischemic stroke or TIA. N Engl J Med.

[92] Wang Y, Wang Y, Zhao X (2013). Clopidogrel with aspirin in acute minor stroke or transient ischemic attack. N Engl J Med.

[93] Chang H, Rockman CB, Jacobowitz GR, Veith FJ, Sadek M, Kashyap VS, Maldonado TS (2021). Statin use reduces mortality in patients who develop major complications after transcarotid artery revascularization. J Vasc Surg.

[94] Chang H, Zeeshan M, Rockman CB (2022). Statin use reduces mortality in patients who develop major complications after transcarotid artery revascularization. Ann Vasc Surg.

[95] Arinze N, Farber A, Sachs T (2018). The effect of statin use and intensity on stroke and myocardial infarction after carotid endarterectomy. J Vasc Surg.

[96] Litsky J, Stilp E, Njoh R, Mena-Hurtado C (2014). Management of symptomatic carotid disease in 2014. Curr Cardiol Rep.

[97] Grotta JC (2013). Clinical practice. Carotid stenosis. N Engl J Med.

[98] Howell SJ (2007). Carotid endarterectomy. Br J Anaesth.

[99] Messas E, Goudot G, Halliday A (2020). Management of carotid stenosis for primary and secondary prevention of stroke: state-of-the-art 2020: a critical review. Eur Heart J Suppl.

[100] AbuRahma AF, Avgerinos ED, Chang RW (2022). Society for Vascular Surgery clinical practice guidelines for management of extracranial cerebrovascular disease. J Vasc Surg.

[101] Brott TG, Calvet D, Howard G (2019). Long-term outcomes of stenting and endarterectomy for symptomatic carotid stenosis: a preplanned pooled analysis of individual patient data. Lancet Neurol.

[102] Müller MD, Lyrer P, Brown MM, Bonati LH (2020). Carotid artery stenting versus endarterectomy for treatment of carotid artery stenosis. Cochrane Database Syst Rev.

[103] White CJ, Brott TG, Gray WA (2022). Carotid artery stenting: JACC state-of-the-art review. J Am Coll Cardiol.

[104] Lal BK, Beach KW, Roubin GS (2012). Restenosis after carotid artery stenting and endarterectomy: a secondary analysis of CREST, a randomised controlled trial. Lancet Neurol.

[105] Spence JD, Song H, Cheng G (2016). Appropriate management of asymptomatic carotid stenosis. Stroke Vasc Neurol.

[106] Brott TG, Meschia JF, Lal BK, Chamorro Á, Howard VJ, Howard G (2023). When will we have what we need to advise patients how to manage their carotid stenosis?: Lessons from SPACE-2. Stroke.

[107] Ferguson GG, Eliasziw M, Barr HW (1999). The North American Symptomatic Carotid Endarterectomy Trial: surgical results in 1415 patients. Stroke.

[108] Barnett HJ, Taylor DW, Eliasziw M (1998). Benefit of carotid endarterectomy in patients with symptomatic moderate or severe stenosis. North American Symptomatic Carotid Endarterectomy Trial Collaborators. N Engl J Med.

[109] Brott TG, Hobson RW 2nd, Howard G (2010). Stenting versus endarterectomy for treatment of carotid-artery stenosis. N Engl J Med.

[110] Eckstein HH, Ringleb P, Allenberg JR (2008). Results of the Stent-Protected Angioplasty versus Carotid Endarterectomy (SPACE) study to treat symptomatic stenoses at 2 years: a multinational, prospective, randomised trial. Lancet Neurol.

[111] Reiff T, Eckstein HH, Mansmann U (2022). Carotid endarterectomy or stenting or best medical treatment alone for moderate-to-severe asymptomatic carotid artery stenosis: 5-year results of a multicentre, randomised controlled trial. Lancet Neurol.

[112] Halliday A, Bulbulia R, Bonati LH, Chester J, Cradduck-Bamford A, Peto R, Pan H (2021). Second asymptomatic carotid surgery trial (ACST-2): a randomised comparison of carotid artery stenting versus carotid endarterectomy. Lancet.

[113] Zhang Y, Bai Y, Xie J, Wang J, He L, Huang M, Xu F (2022). Carotid plaque components and other carotid artery features associated with risk of stroke: a systematic review and meta-analysis. J Stroke Cerebrovasc Dis.

[114] Lovrencic-Huzjan A, Rundek T, Katsnelson M (2012). Recommendations for management of patients with carotid stenosis. Stroke Res Treat.

[115] Flaherty ML, Kissela B, Khoury JC (2013). Carotid artery stenosis as a cause of stroke. Neuroepidemiology.

[116] Altun G, Pulathan Z, Hemsinli D (2018). True aneurysms of the extracranial carotid artery: an evaluation of two "giant aneurysms" and the current literature. J Korean Neurosurg Soc.

[117] Jou LD, Shaltoni HM, Morsi H, Mawad ME (2010). Hemodynamic relationship between intracranial aneurysm and carotid stenosis: review of clinical cases and numerical analyses. Neurol Res.

[118] Ni L, Pu Z, Zeng R, Zhang R, Zheng YH, Ye W, Liu CW (2016). Endovascular stenting for extracranial carotid artery aneurysms: experiences and mid-term results. Medicine (Baltimore).

[119] Sulženko J, Paluszek P, Machnik R, Widimský P, Jarkovský J, Pieniazek P (2019). Prevalence and predictors of coronary artery disease in patients undergoing carotid artery stenting. Coron Artery Dis.

[120] Wanamaker KM, Moraca RJ, Nitzberg D, Magovern GJ Jr (2012). Contemporary incidence and risk factors for carotid artery disease in patients referred for coronary artery bypass surgery. J Cardiothorac Surg.

[121] Steinvil A, Sadeh B, Arbel Y (2011). Prevalence and predictors of concomitant carotid and coronary artery atherosclerotic disease. J Am Coll Cardiol.

[122] Naylor R, Rantner B, Ancetti S (2023). Editor's Choice - European Society for Vascular Surgery (ESVS) 2023 Clinical Practice Guidelines on the management of atherosclerotic carotid and vertebral artery disease. Eur J Vasc Endovasc Surg.

[123] Plessers M, Van Herzeele I, Vermassen F, Vingerhoets G (2014). Neurocognitive functioning after carotid revascularization: a systematic review. Cerebrovasc Dis Extra.

[124] Lopes A, Gomes ML, Sobrinho G, Pedro LM (2020). Surgical treatment of post-carotid endarterectomy carotid pseudoaneurysm. EJVES Short Rep.

[125] Heskett C, Brake A, Fry L (2022). Treatment options for pseudoaneurysm after carotid endarterectomy: a systematic review and illustrative case. World Neurosurg.

[126] Dalio MB, Ribeiro da Silva Filho E, Santarosa MB, Junior TT, Ribeiro MS, Joviliano EE (2021). Transcervical access for endograft exclusion of a postendarterectomy carotid pseudoaneurysm in a patient with type III aortic arch. Vasc Endovascular Surg.

[127] Arquizan C, Trinquart L, Touboul PJ (2011). Restenosis is more frequent after carotid stenting than after endarterectomy: the EVA-3S study. Stroke.

[128] Bonati LH, Dobson J, Featherstone RL (2015). Long-term outcomes after stenting versus endarterectomy for treatment of symptomatic carotid stenosis: the International Carotid Stenting Study (ICSS) randomised trial. Lancet.

[129] Brott TG, Howard G, Roubin GS (2016). Long-term results of stenting versus endarterectomy for carotid-artery stenosis. N Engl J Med.

[130] Bonati LH, Gregson J, Dobson J (2018). Restenosis and risk of stroke after stenting or endarterectomy for symptomatic carotid stenosis in the International Carotid Stenting Study (ICSS): secondary analysis of a randomised trial. Lancet Neurol.

[131] Hirt LS (2014). Progression rate and ipsilateral neurological events in asymptomatic carotid stenosis. Stroke.

[132] Kakkos SK, Nicolaides AN, Charalambous I (2014). Predictors and clinical significance of progression or regression of asymptomatic carotid stenosis. J Vasc Surg.

[133] Kakkos SK, Sabetai M, Tegos T (2009). Silent embolic infarcts on computed tomography brain scans and risk of ipsilateral hemispheric events in patients with asymptomatic internal carotid artery stenosis. J Vasc Surg.

[134] King A, Serena J, Bornstein NM, Markus HS (2011). Does impaired cerebrovascular reactivity predict stroke risk in asymptomatic carotid stenosis? A prospective substudy of the asymptomatic carotid emboli study. Stroke.

